# Targeted Next-Generation Sequencing Reveals Novel *USH2A* Mutations Associated with Diverse Disease Phenotypes: Implications for Clinical and Molecular Diagnosis

**DOI:** 10.1371/journal.pone.0105439

**Published:** 2014-08-18

**Authors:** Xue Chen, Xunlun Sheng, Xiaoxing Liu, Huiping Li, Yani Liu, Weining Rong, Shaoping Ha, Wenzhou Liu, Xiaoli Kang, Kanxing Zhao, Chen Zhao

**Affiliations:** 1 Department of Ophthalmology, The First Affiliated Hospital of Nanjing Medical University, State Key Laboratory of Reproductive Medicine, Nanjing, China; 2 Ningxia Eye Hospital, Ningxia People’s Hospital, Ningxia, China; 3 Department of Ophthalmology, Xinhua Hospital, Shanghai Jiao Tong University School of Medicine, Shanghai, China; 4 Tianjin Medical University, Tianjin Eye Hospital, Tianjin Key Laboratory of Ophthalmology and Visual Science, Tianjin, China; 5 State Key Laboratory of Ophthalmology, Zhongshan Ophthalmic Center, Sun Yat Sen University, Guangzhou, China; Pasteur Institute of Lille, France

## Abstract

*USH2A* mutations have been implicated in the disease etiology of several inherited diseases, including Usher syndrome type 2 (USH2), nonsyndromic retinitis pigmentosa (RP), and nonsyndromic deafness. The complex genetic and phenotypic spectrums relevant to *USH2A* defects make it difficult to manage patients with such mutations. In the present study, we aim to determine the genetic etiology and to characterize the correlated clinical phenotypes for three Chinese pedigrees with nonsyndromic RP, one with RP sine pigmento (RPSP), and one with USH2. Family histories and clinical details for all included patients were reviewed. Ophthalmic examinations included best corrected visual acuities, visual field measurements, funduscopy, and electroretinography. Targeted next-generation sequencing (NGS) was applied using two sequence capture arrays to reveal the disease causative mutations for each family. Genotype-phenotype correlations were also annotated. Seven *USH2A* mutations, including four missense substitutions (p.P2762A, p.G3320C, p.R3719H, and p.G4763R), two splice site variants (c.8223+1G>A and c.8559-2T>C), and a nonsense mutation (p.Y3745*), were identified as disease causative in the five investigated families, of which three reported to have consanguineous marriage. Among all seven mutations, six were novel, and one was recurrent. Two homozygous missense mutations (p.P2762A and p.G3320C) were found in one individual family suggesting a potential double hit effect. Significant phenotypic divergences were revealed among the five families. Three families of the five families were affected with early, moderated, or late onset RP, one with RPSP, and the other one with USH2. Our study expands the genotypic and phenotypic variability relevant to *USH2A* mutations, which would help with a clear insight into the complex genetic and phenotypic spectrums relevant to *USH2A* defects, and is complementary for a better management of patients with such mutations. We have also demonstrated that a targeted NGS approach is a valuable tool for the genetic diagnosis of USH2 and RP.

## Background

Inherited retinal dystrophies (IRDs) comprise a group of monogenic diseases presenting significant clinical and genetic heterogeneities. Retinitis pigmentosa (RP; MIM: 26800), the most common form of IRDs, possesses a global prevalence of 1 in 3500 to 5000 individuals [1]. In the disease course of RP, rod photoreceptors will initially be affected thus leading to night blindness and visual field (VF) constrictions, while subsequent cone defects will cause color blindness, central vision impairments, and finally total vision loss [2].

Clinically, RP can be divided into nonsyndromic and syndromic forms. Former one can be further classified into typical and atypical RP according to the fundus presentations, while the latter one is usually accompanied by systemic abnormalities [2]. Patients affected with RP may manifest great varieties in the disease course, and RP presentations can overlay with other forms of IRDs, thus making it more difficult to obtain better clinical diagnoses for these patients. Genetically, RP, usually a monogenic disorder, demonstrates all three types of mendelian inheritance modes, including autosomal dominant, autosomal recessive, and X-linked patterns. Additionally, digenic inheritance patterns have also been reported [3]. Hitherto, 71 mapped loci involving 64 genes have been identified as disease-causing for nonsyndromic RP (www.retnet.org). The significant genetic heterogeneity calls for an efficient platform for the molecular diagnosis of RP. Targeted next-generation sequencing (NGS) strategies show significant improvements when compared with traditional detection approaches. It is more effective, cost-effective, and provides high sequencing accuracy. Applying NGS approaches to IRDs is now relatively sophisticated. A targeted panel of known IRDs causative genes would help to obtain a potential molecular diagnosis for 50–70% of IRDs cases [4].

**Figure 1:**
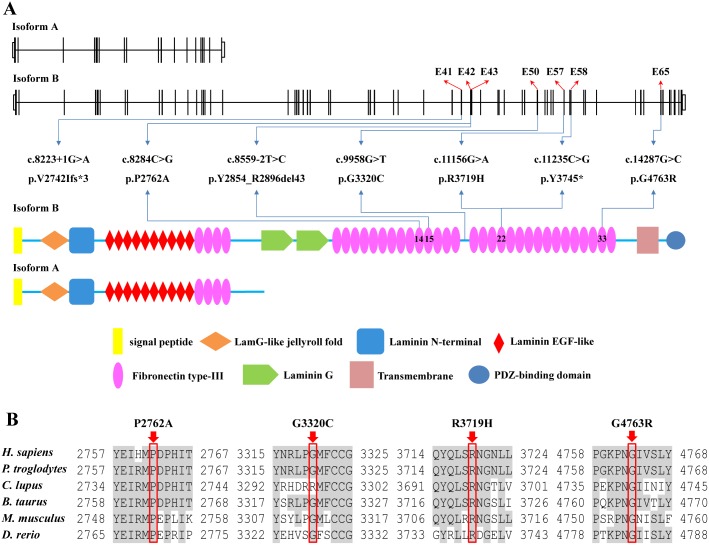
Mutations Identified in the Present Study. (A) The genome and protein structures of the long and short isoforms (isoforms a and b) of *USH2A* are presented. Schematic representation of the relative linear location of all seven *USH2A* mutations identified in the present study in context of the genome structure (upper) and protein structure (below) of isoform b. Domains of the usherin protein are indicated. (B) Evolutionary conservation of the P2762, G3320, R3719 and G4763 residues in the usherin protein of six species.

*USH2A* (MIM: 608400), located on chromosome 1 and sublocation 1q41, encodes the usherin, which demonstrates a wide but not ubiquitous tissue distribution [5]. Usherin shows two alternatively spliced isoforms that comprise of short “isoform a” and longer “isoform b” variants [6], [7], [8]. Isoform a encompasses 21 exons and generates a secreted and extracellular protein of 1546 residues, while isoform b contains 51 additional exons at the C terminal, and encodes a protein with 5202 amino acids (**Figure 1A**). As a basement membrane protein, usherin has been localized to the basement membrane of the retina, cochlea, and a series of other tissues [5], [9], [10]. Despite its wide expression, defects in usherin have only been linked with Usher syndrome type 2 (USH2; MIM: 276901) [11], [12], [13], [14], nonsyndromic RP [15], and nonsyndromic hearing loss (NSHL) [16], implying its special role in maintaining the function of the capillary and structural basement membranes of the two ciliated sensory neurons, namely photoreceptor cells and cochlear hair cells. As indicated by previous studies, usherin is localized to the apical inner segment recess which wraps around the connecting cilia in photoreceptor cells, and is associated with the hair bundles during postnatal development in cochlear hair cells [17]. Although many studies have been focused on the properties of usherin, the mechanism underlying the great phenotypic variety led by *USH2A* mutations has not been well illustrated yet.

Herein, using a targeted NGS approach, we determined the disease causative mutations in three Chinese families with typical autosomal recessive RP (ARRP), one with RP sine pigmento (RPSP), and one with USH2. Six novel and one recurrent homozygous *USH2A* mutations were identified with their genotype-phenotype correlations annotated.

## Methods

### Participants and Clinical Assessments

This study, conformed to the tenets of the Declaration of Helsinki, was approved and prospectively reviewed by the Ethics Committee on Human Research of Nanjing Medical University. Seven patients and seven unaffected family members from five unrelated families with autosomal recessive inheritance pattern of retinal disease were recruited from the Frist Affiliated Hospital of Nanjing Medical University. Written informed consents were signed by all participants before their participation. Consanguineous marriages were reported by three families. Pure tone audiometry testing and vestibular tests were performed on all included patients. Detailed ophthalmic examinations were also conducted, including assessments of best corrected visual acuities (BCVAs), VF measurements, funduscopy, electroretinography (ERG), and fundus fluorescein angiography (FFA). Optical coherence tomography (OCT) examination was selectively conducted on patients with macular involvements. Peripheral blood samples were collected using 5 mL tubes containing ethylenediamantetetraacetic acid (EDTA) from all participants and 150 additional negative controls free of retinal or other ocular diseases. Genomic DNA was isolated from leukocytes with a QIAmp DNA blood kit (Qiagen, Valencia, CA) in accordance to the manufacturer’s protocols, and stored at −20°C before used.

### Targeted NGS Approach and Bioinformatics Analysis

A targeted NGS approach was conducted on patients ARRP01-IV:4, ARRP02-II:3, ARRP03-IV:3, and ARRP04-IV:4 using a microarray that targeted 180 IRDs causative and 9 candidate genes, while on patient SU-II:2 using a different microarray that targeted 319 ophthalmic disease relevant genes. Both microarrays were designed to capture the coding sequences, 100 base pairs (bp) flanking regions, together with 5′- and 3′-untranslated regions (UTR) of the selected targeted genes. Detailed information of the two microarrays has been previously described [1], [18], [19], [20]. Library preparation, qualification, and NGS on the Illumina HiSeq2000 platform (Illumina, Inc., San Diego, CA, USA) were performed in collaboration with BGI-Shenzhen (Shenzhen, Guangdong, China) per the manufacturer’s instructions [18]. Bioinformatics analysis was conducted as detailed previously [18]. Reads were aligned to the NCBI human reference genome (NCBI build 37.1) for SNP analysis with Short Oligonucleotide Alignment Program (SOAP; http://soap.genomics.org.cn) [21], and for Indel detection using Burrows-Wheeler Aligner (BWA; http://bio-bwa.sourceforge.net/) [22]. Coverage and depth calculations were further performed. All detected variants were filtered against the following 5 SNP databases, including dbSNP137 (http://hgdownload.cse.ucsc.edu/goldenPath/hg19/database/snp137.txt.gz.), HapMap Project (ftp://ftp.ncbi.nlm.nih.gov/hapmap), 1000 Genome Project (ftp://ftp.1000genomes.ebi.ac.uk/vol1/ftp), YH database (http://yh.genomics.org.cn/), and Exome Variant Server (http://evs.gs.washington.edu/EVS/).

### Mutation Verification and *In Silico* Analyses

Variants post-filtration were subsequently verified in all attainable family members. Sanger sequencing was used for mutation verification and prevalence testing in 150 unrelated controls as previously indicated [23]. Primer information for confirmation of *USH2A* mutations is provided in **Table S1**. We further applied Vector NTI Advance 11 software (Invitrogen, Grand Island, NY) to evaluate the evolutionary conservation of the mutated residues by aligning the orthologous sequences of usherin in the following species, including human (*Homo sapiens*; NP_996816.2), chimpanzee (*Pan troglodytes*; XP_514197.2), dog (*Canis lupus familiaris*; XP_545710.2), cow (*Bos taurus*; NP_001178354.1), mouse (*Mus musculus*; NP_067383.3), and zebrafish (*Danio rerio*; XP_697435.4). The potential impacts of the mutation were also predicted using online software, including Sorting Intolerant From Tolerant (SIFT; http://sift.jcvi.org/) [24], Polymorphism Phenotyping v2 (PolyPhen-2, v.2.2.2; http://genetics.bwh.harvard.edu/pph2/) [25], Consensus Deleteriousness score of missense SNVs (CONDEL; http://bg.upf.edu/condel/home) [26], and Protein Variation Effect Analyzer (PROVEAN, v.1.1.3; http://provean.jcvi.org/index.php) [27]. Compute pI/Mw is applied for the computation of the theoretical isoelectric point (http://web.expasy.org/compute_pi/) [28]. The putative splicing alterations caused by splice site mutations were predicted using the following websites, including Human Splicing Finder (v.2.4.1; http://www.umd.be/HSF/) [29], NetGene2 Server (http://www.cbs.dtu.dk/services/NetGene2/) [30], [31], and SplicePort (http://spliceport.cbcb.umd.edu/SplicingAnalyser.html) [32].

## Results

### Targeted NGS Approach

**Figure 2:**
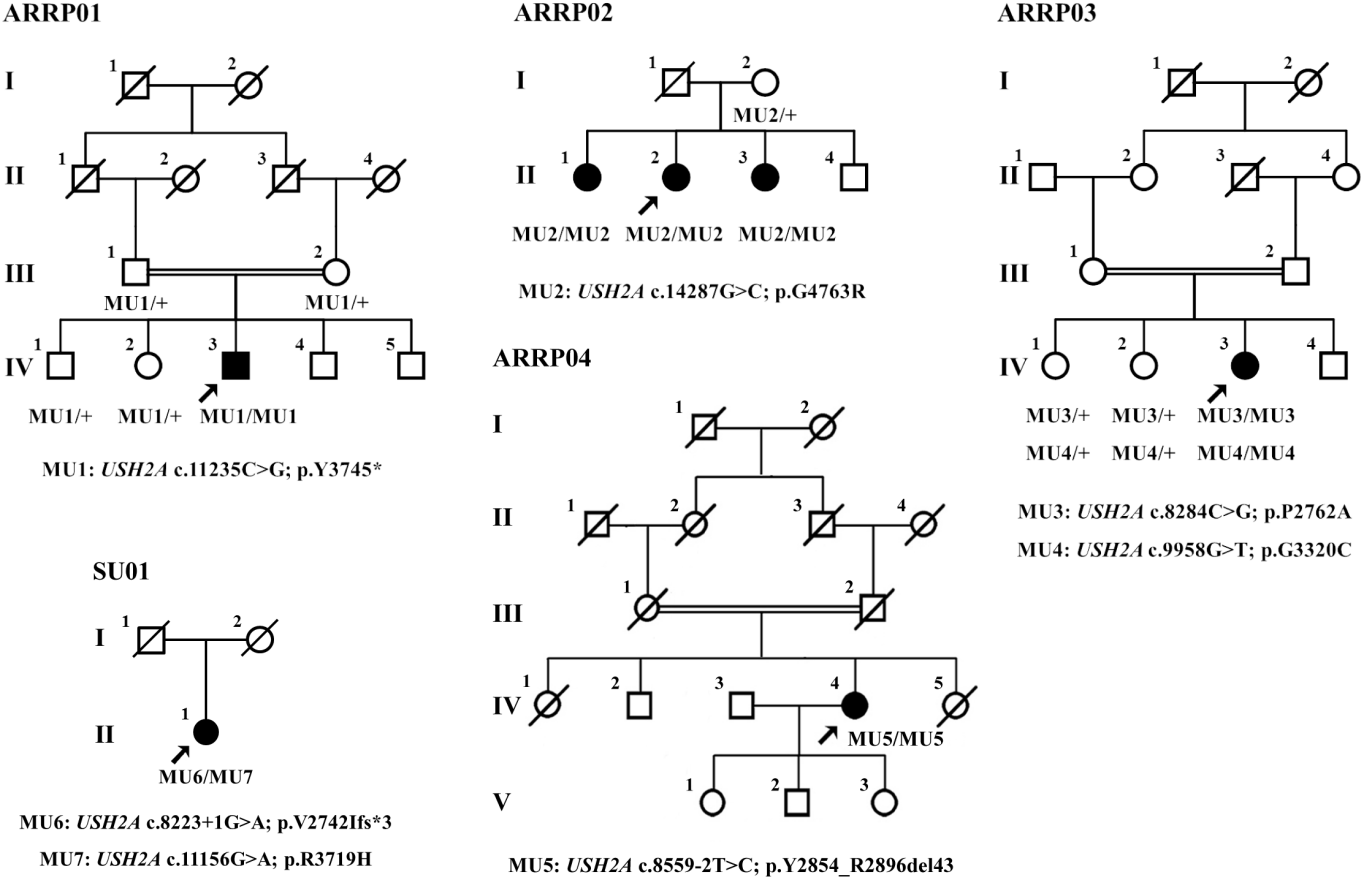
Pedigree Structures of All Five Included Families. Pedigrees of families ARRP01, ARRP02, ARRP03, ARRP04 and SU01 are demonstrated. Consanguineous marriage (double line) was revealed in families ARRP01, ARRP03 and ARRP04. Genotypes of *USH2A* variations for all include members from each individual family are shown with the specific variation detailed under the family pedigree. Arrows indicate probands in each family. Circles indicate females, and squares, males. Filled symbols indicate affected patients, and empty symbols, normal controls.

Seven affected patients from five individual families were included in the present study with their ophthalmic and syndromic evaluations detailed in **Figure 2** and **Table 1**. Targeted NGS approach was selectively conducted on patients ARRP01-IV:4, ARRP02-II:3, ARRP03-IV:3, ARRP04-IV:4, and SU01-II:1, and was shown in **Table S2**. Briefly, coverage of the targeted region was equal to or over 98.55% for each sample, and the average mean depth of five samples was 98.62-fold. Over 2600 variants were initially detected for each sample. After initial filtration, 5, 7, 5, 1, and 11 variations were retained for patients ARRP01-IV:4, ARRP02-II:3, ARRP03-IV:3, ARRP04-IV:4, and SU01-II:2, respectively (**Table 2**). Sanger sequencing further confirmed seven *USH2A* variants as putative disease-causing mutations for the five included families (**Figure 2**). Noteworthy, two homozygous missense variations were revealed as RP causative for patient ARRP03-IV:3, implying a putative double hit effect. The seven identified mutations included a nonsense variation, two splice site variants, and four missense mutations (**Table 3**), all of which were absent in 150 unrelated controls. The conservations of identified variants were detailed in **Figure 1B** with predictions from the four online software programs listed in **Table 3**.

**Table 1:** Clinical features of included patients.

Patient ID	Onset Age(year)	Age(year)/Sex	BCVA(logMAR)		Fundus Appearance								ERG	VF		HearingImpairment	VestibularFunction
			O.D.	O.S.	O.D.				O.S.					O.D.	O.S.		
					MD	OD	AA	PD	MD	OD	AA	PD					
ARRP01-IV:3	13	24/F	0.2	0.3	Severe	Waxy	Yes	Yes	Severe	Waxy	Yes	Yes	NA	<25°	<25°	No	Normal
ARRP02-II:1	22	50/F	0.5	0.5	Mild	Waxy	Yes	Yes	Mild	Waxy	Yes	Yes	Diminished	<45°	<40°	No	Normal
ARRP02-II:2	21	47/F	0.5	0.6	Mild	Waxy	Yes	Yes	Mild	Waxy	Yes	Yes	Diminished	<50	<45°	No	Normal
ARRP02-II:3	17	43/F	0.4	0.5	Mild	Waxy	Yes	Yes	Mild	Waxy	Yes	Yes	Diminished	<30°	<30°	No	Normal
ARRP03-IV:3	12	21/F	0.4	0.5	Mild	Waxy	Yes	No	Mild	Waxy	Yes	No	SignificantlyReduced	<25°	<20°	No	Normal
ARRP04-IV:4	40	62/F	0.6	0.5	Mild	Waxy	Yes	Yes	Mild	Waxy	Yes	Yes	NA	NA	NA	No	Normal
SU01-II:1	5	63/F	LP	LP	Severe	Waxy	Yes	Yes	Severe	Waxy	Yes	Yes	NA	NA	NA	Yes*	Normal

**Abbreviations:** BCVA: best corrected visual acuity; O.D.: right eye; O.S.: left eye; LP: light perception; MD: macular degeneration; OD: optic disk; AA: artery attenuation; PD: pigment deposits; ERG: electroretinography; VF: visual field.

*This patient reported to have hearing impairments since born.

**Table 2:** Variations identified in each individual.

Number	Patient ID				
	ARRP01-IV:3	ARRP02-II:3	ARRP03-IV:3	ARRP04-IV:4	SU01-II:1
**Total Variations**	2638	2652	2624	2732	4113
* SNVs*	2349	2357	2338	2401	3752
* Indels*	289	295	286	331	361
**Post-filtration Variations**	5	7	5	1	11
* SNVs*	5	6	5	1	10
* Indels*	0	1	0	0	1

Abbreviations: SNV: single nucleotide; Indels: insertions and deletions.

**Table 3:** Characteristics of identified *USH2A* mutations.

Family ID	Disease	Variation				Exon/Intron	Bioinformatics Analysis				Reported/Novel	Frequencyin controls
		Nucleotide	Amino Acid	Type	Status		SIFT	PolyPhen	CONDEL	PROVEN		
ARRP01	RP	c.11235C>G	p.Y3745*	nonsense	Hom	Exon 58	NA (/)	NA (/)	NA (/)	NA (/)	Novel	0/300
ARRP02	RP	c.14287G>C	p.G4763R	missense	Hom	Exon 65	DA (0.00)	PD (1.000)	DE (1.000)	DE (−5.10)	Novel	0/300
ARRP03	RPSP	c.8284C>G	p.P2762A	missense	Hom	Exon 42	T (0.19)	B (0.235)	DE (0.500)	DE (−4.56)	Novel	0/300
		c.9958G>T	p.G3320C	missense	Hom	Exon 50	DA (0.00)	PD (1.000)	DE (0.944)	DE (−5.08)	Novel	0/300
ARRP04	RP	c.8559-2T>C	p.Y2854_R2896del43	splicesite	Hom	Intron 42	NA (/)	NA (/)	NA (/)	NA (/)	Reported inUSH2	0/300
SU01	USH2	c.8223+1G>A	p.V2742Ifs*3	splicesite	Het	Intron 41	NA (/)	NA (/)	NA (/)	NA (/)	Novel	0/300
		c.11156G>A	p.R3719H	missense	Het	Exon 57	T (0.14)	PD (1.000)	DE (0.849)	DE (−3.51)	Novel	0/300

Abbreviations: RP: retinitis pigmentosa; RPSP: RP sine pigmento; USH2: usher syndrome type 2; Hom: homozygous; Het: heterozygous; NA: not available; DA: damaging; T: tolerated; PD: probably damaging; B: benign; DE: deleterious.

### Homozygous Mutations Identified in the Three Families with nonsyndromic RP

**Figure 3:**
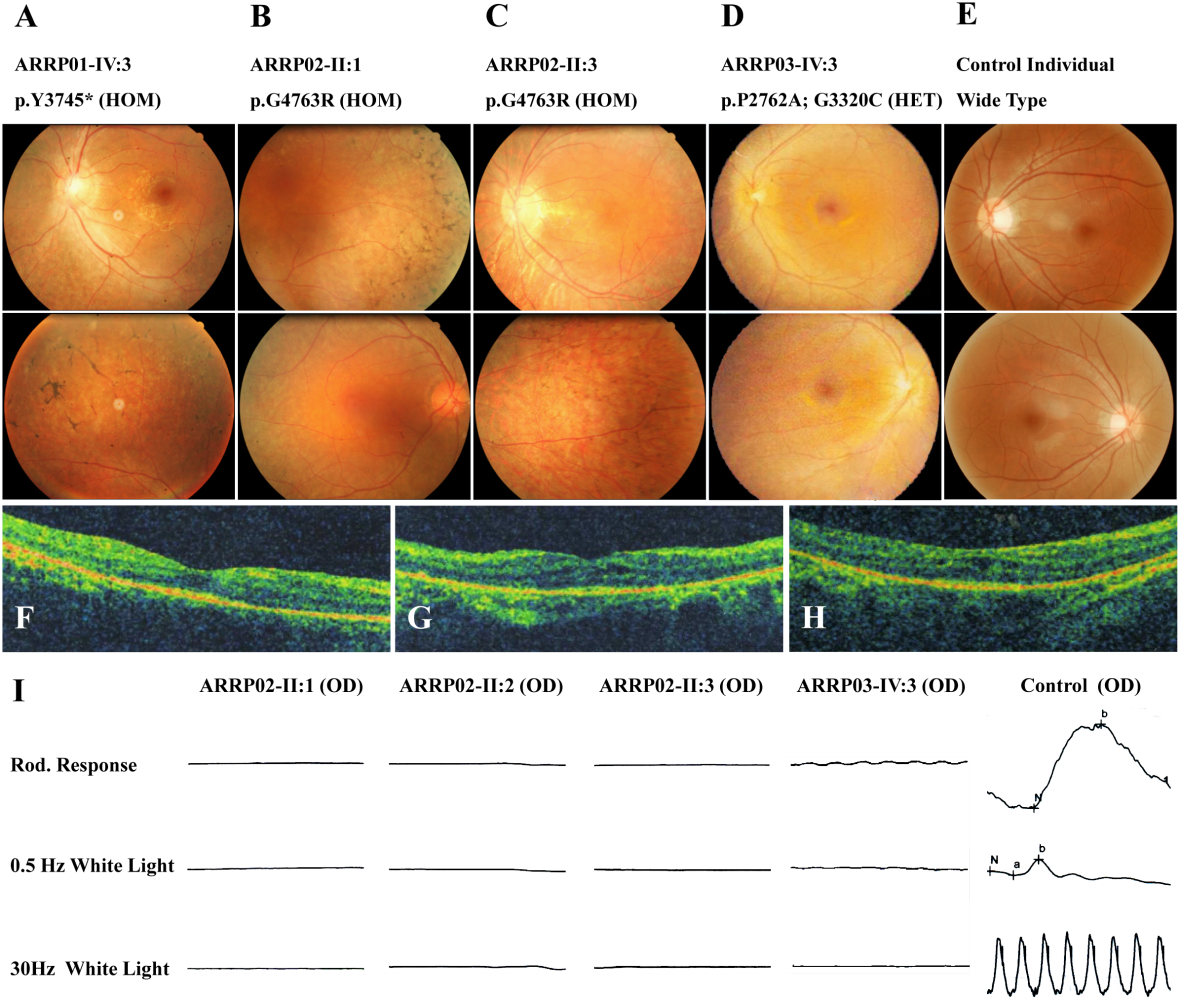
Fundus Appearances of Included Patients. (A) Fundus photos of patient ARRP01-IV:3 demonstrate typical RP presentations, including attenuated vessels, waxy optic disk, and pigment deposits in the mid-peripheral of the retina. Macular degeneration was also identified in this patient. (B–C) Arterial attenuation, waxy of the optic disk, and bone spicular pigmentation are also revealed in the fundus of patients ARRP02-II:1 (B) and ARRP02-II:3 (C), while the macular regions are relatively preserved in both eyes of the two patients. (D) Attenuated arterials, waxy optic discs, and macular degenerations are found in the fundus of patient ARRP03-IV:3. No pigmentations are indicated. (E) Fundus of a control individual. (F–G) The optical coherence tomography (OCT) presentations of patients ARRP02-II:1 (E), ARRP02-II:2 (F) and ARRP02-II:3 (G) all revealed attenuated outer nuclear layer (ONL), retinal pigmented epithelium (RPE), and loss of outer/inner segments (IS/OS). (H) Scotopic and photopic ERG responses for patients ARRP02-II:1, II:2, and II:3 are diminished, while significantly reduced for patient ARRP03-IV:3. ERG presentations of a control individual are also demonstrated.

Patient ARRP01-IV:3 carried a novel homozygous nonsense mutation, c.11235C>G (**Figure 2**). Consanguineous marriage was reported in this family. Patient ARRP01-IV:3 suffered from poor night vision in her early 10 s, which was relatively early when compared with other patients investigated in this study. The disease progressed fast that she had tubular vision at her late 10 s. She was 24 years old at her last visit to our hospital and she complained about her rapidly decreased visual acuities. Macular involvement was also indicated by her fundus appearance (**Figure 3A**).

A novel homozygous missense mutation, c.14287G>C, was identified in all three patients from family ARRP02 (**Figure 2**). This alteration would lead to an amino acid change from a neutral glycine to an alkaline arginine at residue 4763 (p.G4763R) of usherin, thus changing the theoretical isoelectric point from 6.40 to 6.42. The alteration of the isoelectric point of the protein might contribute to the change of the protein function. The three patients were manifested with more moderate ophthalmic phenotypes when compared with patient ARRP01-IV:3. Among all three affected siblings, the proband, patient ARRP02-II:3, got the youngest RP onset age and fastest disease progression (**Table 1**). She complained about her narrowed VF since her late 20 s, and central vision defects since her early 30 s. Typical RP fundi (**Figure 3B**), affected OCT presentations (**Figure 3F–H**), and diminished ERG (**Figure 3I**) were revealed in all three patients.

The mutation found in patient ARRP04-IV:4 was a recurrent one, c.8559-2T>C (**Figure 2**). She was first diagnosed with RP at age 45 in a local hospital. She claimed to have nyctalopia since age 40 and constricted VF since age 43. Slit-lamp examination revealed mild cataracts in her both eyes. Consanguinity was reported. No hearing impairments, vestibular functional abnormalities, or other syndromic defects were observed in patients from the three abovementioned families, thus leading to the clinical diagnosis of nonsyndromic RP.

### Mutations in Patient ARRP03-IV:3 with RPSP

Another two novel homozygous missense mutations, c.8284C>G and c.9958G>T, were found in patient ARRP03-IV:3 (**Figure 2**). The former one leads to an amino acid change from a proline to an alanine at residue 2762 (p.P2762A) of usherin, while the latter one causes the alteration from glycine to cystine at position 3320 (p.G3320C). Patient ARRP03-IV:3 showed similar RP progress to that of patient ARRP01-IV:3. She reported to have nyctalopia at age 12 and constricted VF since age 14. Significantly reduced ERG presentations were also indicated (**Figure 3I**). Attenuated vessels, waxy optic disc, and moderate macular atrophy were also revealed by funduscopy, while no pigment deposits were identified (**Figure 3D**), thus leading to the clinical diagnosis of RPSP. No systemic defects were identified. Consanguineous marriage was indicated.

### Mutations Validated in Patient SU01-II:1 with USH2

Heterozygous novel mutations, c.8223+1G>A and c.11156G>A, were found in patient SU01-II:1 (**Figure 2**). The former one was a single nucleotide change that might affect splicing of *USH2A*, while the second mutation was a missense variant, which would lead to the substitution of an arginine to a histidine at residue 3719 of usherin. Patient SU01-II:1 reported to have congenital and stable hearing impairments. No vestibular defect was identified. Typical RP presentations were also revealed, that she complained of night blindness since age 5, youngest among all included patients. Her clinical symptoms supported the clinical diagnosis of USH2.

## Discussion

*USH2A* mutations account for over 70% of USH2 cases in multiple populations [11], [12], [13], [14]. Recent studies have revealed the important role of *USH2A* mutations in causing nonsydromic RP [15] and NSHL [16], indicating a wide phenotypic variety mediated by *USH2A* mutations. In this study, we report six novel and one recurrent mutations in the *USH2A* gene as the disease-causing mutations for five families. Diverse clinical phenotypes, including early, moderate, and late onset non-syndromic RP, RPSP, and USH2, have been shown, supporting the complex genotype-phenotype correlations for *USH2A* mutations. Noteworthy, a previously identified founder mutation for USH2 in East Asian populations [13], [14], [33], c.8559-2T>C, was found correlated to late onset RP in the present study, further suggesting the complexity. Meanwhile, to the best of our knowledge, this is the first time that RPSP is found associated with *USH2A* mutations, and the two identified homozygous *USH2A* mutations suggest a potential double hit effect.

Of all the seven identified mutations, four are missense substitutions, while the other three would generate truncated proteins or lead to nonsense mediated mRNA decay (NMD), including c.8223+1G>A found in family SU01, c.8559-2T>C in family ARRP04, and c.11235C>G in family ARRP01. The mutation c.8223+1G>A would probably affect a consensus donor splice site essential for splicing of intron 41. All four online programs predicted that this mutation would abolish the original splice donor site, leading to the insertion of intron 41. However, a premature termination codon (PTC) located at c.8223+10 to c.8223+12 in intron 41, TGA, would suspend translation, leading to NMD and/or the generation of a truncated protein, p.V2742Ifs*3. The sever impacts of this mutation may partly help to explain for the comparatively earlier onset of her clinically manifestations. The mutation c.8559-2T>C, located at the splice acceptor site in intron 42, is recognized as a frequent mutation for USH2 in Asian populations [13], [14], [33], but has never been reported in causing RP. This mutation is proved to cause the skipping of *USH2A* exon 43 by reverse-transcription PCR using patients’ hair roots thus leading to the elimination of 43 amino acids in usherin (p.Y2854_R2896del43) [10]. The nonsense mutation, c.11235C>G, would generate a PTC, and further lead to dysfunction or a reduction of usherin through truncation of the generated protein or NMD, respectively.

Two different forms of usherin have been revealed. The shorter isoform, isoform a, contains five domains, including an N-terminal signal peptide, laminin G-like jellyroll fold, laminin N-terminal, laminin-type EGF-like modules, and fibronectin type III repeats. The longer isoform, isoform b, contains additional domains, including laminin G domains, a transmembrane region, and a C-terminal PSD-95/Discs Large/Zona Occludens 1 (PDZ)-binding motif, via which this form is attached to the plasma membrane of retinal photoreceptors [17]. Isoform b can therefore be divided into three major parts, namely the extracellular region, the transmembrane domain, and the cytoplasmic part. Isoform b has been proved as the predominant form in the retina, and is crucial for the maintenance of retinal photoreceptor cells and the development of cochlear hair cells [17]. For mutations located within the genome context of isoform a, it can usually affect the function of isoforms a and b. All seven mutations identified in the present study locate within isoform b, among which, p.Y3745* in family ARRP01, p.G4763R in family ARRP02, p.P2762A in family ARRP03, p.Y2854_R2896del43 in family ARRP04, and p.R3719H in family SU01, locate within the fibronectin type-III repeats, while another mutation in family ARRP04, p.G3320C, was found in the interval between the 18^th^ and the 19^th^ fibronectin type-III domains (**Figure 1A**). However, the expression level of the long isoform is low in the cochlea [7], which could possibly help to explain the absence of hearing and vestibular impairments in some patients with *USH2A* mutations [15], [34], [35]. Previous studies have also hypothesized a potential correlation between the nature of the variants and clinical phenotypes, indicating that non-syndromic RP patients would carry *USH2A* mutations with less perniciousness, or with deleterious impacts in only one allele [34], [36], which may also help to account for the diverse clinical phenotypes as demonstrated in the present study.

Herein, we have identified six novel and one recurrent *USH2A* mutations using targeted NGS approaches, which further demonstrates that targeted NGS approaches are valuable tools for genetic diagnoses. However, many limitations still exist that prevent the routine application of targeted NGS in the clinic, including cost, infrastructural logistics, strategies for patient selection, and interpretation of genetic datasets [4]. Among all, interpretation of genetic datasets is most important while also most problematic [4]. Bioinformatics predictions are useful, but the results can sometimes be confusing or even incorrect [37]. Therefore, more functional analyses are needed to confirm the pathogenesis of distinct mutations, and also to gain a clearer insight into the mechanism underlying the selectively clinical phenotypes caused by different mutations.

In conclusion, our findings highlight the genotypic multiplicity and phenotypic variability for *USH2A* mutations. A better understanding of the genotype-phenotype correlations would help with a better management for patients with *USH2A* mutations, provide thorough viewpoints into the pathogenesis of *USH2A* defects, and assist with the development of putative therapeutic approaches.

## Supporting Information

Table S1 **Primer information for confirmation of USH2A mutations.**
(DOC)

Table S2 **Overview of data production.**
(DOC)

## References

[1] Chen X, Liu Y, Sheng X, Tam PO, Zhao K, et al. (2014) PRPF4 mutations cause autosomal dominant retinitis pigmentosa. Hum Mol Genet. 10.1093/hmg/ddu005 24419317

[2] Hartong DT, Berson EL, Dryja TP (2006) Retinitis pigmentosa. Lancet 368: 1795–1809. 17113430 10.1016/S0140-6736(06)69740-7

[3] Kajiwara K, Berson EL, Dryja TP (1994) Digenic retinitis pigmentosa due to mutations at the unlinked peripherin/RDS and ROM1 loci. Science 264: 1604–1608. 8202715 10.1126/science.8202715

[4] Davies WI (2014) Challenges using diagnostic next-generation sequencing in the clinical environment for inherited retinal disorders. Pers Med 11: 99–111. 10.2217/pme.13.95 29751394

[5] Bhattacharya G, Miller C, Kimberling WJ, Jablonski MM, Cosgrove D (2002) Localization and expression of usherin: a novel basement membrane protein defective in people with Usher’s syndrome type IIa. Hear Res 163: 1–11. 11788194 10.1016/s0378-5955(01)00344-6

[6] Weston MD, Eudy JD, Fujita S, Yao S, Usami S, et al (2000) Genomic structure and identification of novel mutations in usherin, the gene responsible for Usher syndrome type IIa. Am J Hum Genet 66: 1199–1210. 10729113 10.1086/302855 PMC1288187

[7] van Wijk E, Pennings RJ, te Brinke H, Claassen A, Yntema HG, et al (2004) Identification of 51 novel exons of the Usher syndrome type 2A (USH2A) gene that encode multiple conserved functional domains and that are mutated in patients with Usher syndrome type II. Am J Hum Genet 74: 738–744. 15015129 10.1086/383096 PMC1181950

[8] Eudy JD, Weston MD, Yao S, Hoover DM, Rehm HL, et al (1998) Mutation of a gene encoding a protein with extracellular matrix motifs in Usher syndrome type IIa. Science 280: 1753–1757. 9624053 10.1126/science.280.5370.1753

[9] Pearsall N, Bhattacharya G, Wisecarver J, Adams J, Cosgrove D, et al (2002) Usherin expression is highly conserved in mouse and human tissues. Hear Res 174: 55–63. 12433396 10.1016/s0378-5955(02)00635-4

[10] Nakanishi H, Ohtsubo M, Iwasaki S, Hotta Y, Mizuta K, et al (2010) Hair roots as an mRNA source for mutation analysis of Usher syndrome-causing genes. J Hum Genet 55: 701–703. 20596040 10.1038/jhg.2010.83

[11] Le Quesne Stabej P, Saihan Z, Rangesh N, Steele-Stallard HB, Ambrose J, et al (2012) Comprehensive sequence analysis of nine Usher syndrome genes in the UK National Collaborative Usher Study. J Med Genet 49: 27–36. 22135276 10.1136/jmedgenet-2011-100468 PMC3678402

[12] Bonnet C, Grati M, Marlin S, Levilliers J, Hardelin JP, et al (2011) Complete exon sequencing of all known Usher syndrome genes greatly improves molecular diagnosis. Orphanet J Rare Dis 6: 21. 21569298 10.1186/1750-1172-6-21 PMC3125325

[13] Dai H, Zhang X, Zhao X, Deng T, Dong B, et al (2008) Identification of five novel mutations in the long isoform of the USH2A gene in Chinese families with Usher syndrome type II. Mol Vis 14: 2067–2075. 19023448 PMC2584772

[14] Nakanishi H, Ohtsubo M, Iwasaki S, Hotta Y, Usami S, et al (2011) Novel USH2A mutations in Japanese Usher syndrome type 2 patients: marked differences in the mutation spectrum between the Japanese and other populations. J Hum Genet 56: 484–490. 21593743 10.1038/jhg.2011.45

[15] Bernal S, Ayuso C, Antinolo G, Gimenez A, Borrego S, et al (2003) Mutations in USH2A in Spanish patients with autosomal recessive retinitis pigmentosa: high prevalence and phenotypic variation. J Med Genet 40: e8. 12525556 10.1136/jmg.40.1.e8 PMC1735247

[16] Mutai H, Suzuki N, Shimizu A, Torii C, Namba K, et al (2013) Diverse spectrum of rare deafness genes underlies early-childhood hearing loss in Japanese patients: a cross-sectional, multi-center next-generation sequencing study. Orphanet J Rare Dis 8: 172. 24164807 10.1186/1750-1172-8-172 PMC4231469

[17] Liu X, Bulgakov OV, Darrow KN, Pawlyk B, Adamian M, et al (2007) Usherin is required for maintenance of retinal photoreceptors and normal development of cochlear hair cells. Proc Natl Acad Sci USA 104: 4413–4418. 17360538 10.1073/pnas.0610950104 PMC1838616

[18] Chen X, Zhao K, Sheng X, Li Y, Gao X, et al (2013) Targeted sequencing of 179 genes associated with hereditary retinal dystrophies and 10 candidate genes identifies novel and known mutations in patients with various retinal diseases. Invest Ophthalmol Vis Sci 54: 2186–2197. 23462753 10.1167/iovs.12-10967

[19] Rong W, Chen X, Zhao K, Liu Y, Liu X, et al (2014) Novel and recurrent MYO7A mutations in Usher syndrome type 1 and type 2. PLoS One 9: e97808. 24831256 10.1371/journal.pone.0097808 PMC4022727

[20] Pan X, Chen X, Liu X, Gao X, Kang X, et al (2014) Mutation analysis of pre-mRNA splicing genes in Chinese families with retinitis pigmentosa. Mol Vis 20: 770–779. 24940031 PMC4043610

[21] Li R, Yu C, Li Y, Lam TW, Yiu SM, et al (2009) SOAP2: an improved ultrafast tool for short read alignment. Bioinformatics 25: 1966–1967. 19497933 10.1093/bioinformatics/btp336

[22] Li H, Durbin R (2010) Fast and accurate long-read alignment with Burrows-Wheeler transform. Bioinformatics 26: 589–595. 20080505 10.1093/bioinformatics/btp698 PMC2828108

[23] Zhao C, Lu S, Zhou X, Zhang X, Zhao K, et al (2006) A novel locus (RP33) for autosomal dominant retinitis pigmentosa mapping to chromosomal region 2cen-q12.1. Hum Genet 119: 617–623. 16612614 10.1007/s00439-006-0168-3

[24] Kumar P, Henikoff S, Ng PC (2009) Predicting the effects of coding non-synonymous variants on protein function using the SIFT algorithm. Nat Protoc 4: 1073–1081. 19561590 10.1038/nprot.2009.86

[25] Adzhubei IA, Schmidt S, Peshkin L, Ramensky VE, Gerasimova A, et al (2010) A method and server for predicting damaging missense mutations. Nat Methods 7: 248–249. 20354512 10.1038/nmeth0410-248 PMC2855889

[26] Gonzalez-Perez A, Lopez-Bigas N (2011) Improving the assessment of the outcome of nonsynonymous SNVs with a consensus deleteriousness score, Condel. Am J Hum Genet 88: 440–449. 21457909 10.1016/j.ajhg.2011.03.004 PMC3071923

[27] Choi Y, Sims GE, Murphy S, Miller JR, Chan AP (2012) Predicting the functional effect of amino acid substitutions and indels. PLoS One 7: e46688. 23056405 10.1371/journal.pone.0046688 PMC3466303

[28] Bjellqvist B, Basse B, Olsen E, Celis JE (1994) Reference points for comparisons of two-dimensional maps of proteins from different human cell types defined in a pH scale where isoelectric points correlate with polypeptide compositions. Electrophoresis 15: 529–539. 8055880 10.1002/elps.1150150171

[29] Desmet FO, Hamroun D, Lalande M, Collod-Beroud G, Claustres M, et al (2009) Human Splicing Finder: an online bioinformatics tool to predict splicing signals. Nucleic Acids Res 37: e67. 19339519 10.1093/nar/gkp215 PMC2685110

[30] Hebsgaard SM, Korning PG, Tolstrup N, Engelbrecht J, Rouze P, et al (1996) Splice site prediction in Arabidopsis thaliana pre-mRNA by combining local and global sequence information. Nucleic Acids Res 24: 3439–3452. 8811101 10.1093/nar/24.17.3439 PMC146109

[31] Brunak S, Engelbrecht J, Knudsen S (1991) Prediction of human mRNA donor and acceptor sites from the DNA sequence. J Mol Biol 220: 49–65. 2067018 10.1016/0022-2836(91)90380-o

[32] Dogan RI, Getoor L, Wilbur WJ, Mount SM (2007) SplicePort–an interactive splice-site analysis tool. Nucleic Acids Res 35: W285–291. 17576680 10.1093/nar/gkm407 PMC1933122

[33] Nakanishi H, Ohtsubo M, Iwasaki S, Hotta Y, Mizuta K, et al (2009) Identification of 11 novel mutations in USH2A among Japanese patients with Usher syndrome type 2. Clin Genet 76: 383–391. 19737284 10.1111/j.1399-0004.2009.01257.x

[34] Mendez-Vidal C, Gonzalez-Del Pozo M, Vela-Boza A, Santoyo-Lopez J, Lopez-Domingo FJ, et al (2013) Whole-exome sequencing identifies novel compound heterozygous mutations in USH2A in Spanish patients with autosomal recessive retinitis pigmentosa. Mol Vis 19: 2187–2195. 24227914 PMC3820429

[35] McGee TL, Seyedahmadi BJ, Sweeney MO, Dryja TP, Berson EL (2010) Novel mutations in the long isoform of the USH2A gene in patients with Usher syndrome type II or non-syndromic retinitis pigmentosa. J Med Genet 47: 499–506. 20507924 10.1136/jmg.2009.075143 PMC3070405

[36] Kaiserman N, Obolensky A, Banin E, Sharon D (2007) Novel USH2A mutations in Israeli patients with retinitis pigmentosa and Usher syndrome type 2. Arch Ophthalmol 125: 219–224. 17296898 10.1001/archopht.125.2.219

[37] Davies WI, Downes SM, Fu JK, Shanks ME, Copley RR, et al (2012) Next-generation sequencing in health-care delivery: lessons from the functional analysis of rhodopsin. Genet Med 14: 891–899. 22791210 10.1038/gim.2012.73

