# Supplementary material for: Targeted Next-Generation Sequencing Reveals Novel USH2A Mutations Associated with Diverse Disease Phenotypes: Implications for Clinical and Molecular Diagnosis
Source: PLoS One. 2014 Aug 18;9(8):e105439. doi: 10.1371/journal.pone.0105439 (PMC4136877; doi:10.1371/journal.pone.0105439)
Supplement: Table S1 — Primer information for confirmation of USH2A mutations. (DOC) [file pone.0105439.s001.doc]

| **Table S1. Primer information for confirmation of *USH2A* mutations.** | | | | |
| --- | --- | --- | --- | --- |
| **Family ID** | **Variation** | **Forward primer (****5'→3')** | **Reverse primer (5'→3')** | **Product length** |
| ARRP01 | c.11235C>G | CAATGGCTTGGAGACAATGTT | CCTGGTGGTATCCAAGCTACA | 343 bp |
| ARRP02 | c.14287G>C | GGTCTGGGCAGTGAATTCTG | CCACCTTTAGCATCCCTCTC | 438 bp |
| ARRP03 | c.8284C>G | GCAAAATTCTAGGCCTCGTG | GCAAGCAACAATGGTGACAG | 245 bp |
|  | c.9958G>T | CCGTACTCCACCTCAGGAAA | TGGAGGACATGACCTTTTCA | 385 bp |
| ARRP04 | c.8559-2T>C | GATGTTATTCTTTATGCTTCCAC | TCACCTGCTAAGACCCTTATCTTC | 250 bp |
| SU01 | c.8223+1G>A | TTCATGGAGGCACAAACAGC | ACACAGAGTCAATCCAGGGT | 174 bp |
|  | c.11156G>A | TCTGCCAGAAAAGCCCAATG | ACAAATCCTGCTGTATGATTGGT | 166 bp |
