# Supplementary material for: Targeted Next-Generation Sequencing Reveals Novel USH2A Mutations Associated with Diverse Disease Phenotypes: Implications for Clinical and Molecular Diagnosis
Source: PLoS One. 2014 Aug 18;9(8):e105439. doi: 10.1371/journal.pone.0105439 (PMC4136877; doi:10.1371/journal.pone.0105439)
Supplement: Table S2 — Overview of data production. (DOC) [file pone.0105439.s002.doc]

| **Table S2. Overview of data production** | | | | | |
| --- | --- | --- | --- | --- | --- |
| **Items** | **ARRP01-IV:3** | **ARRP02-II:3** | **ARRP03-IV:3** | **ARRP04-IV:4** | **SU01-II:1** |
| *Target region (bp)* | 1381060 | 1381060 | 1381060 | 1381060 | 1488489 |
| *Raw reads* | 2195150 | 2465504 | 4397244 | 4441082 | 9996618 |
| *Raw data yield (Mb)* | 198 | 222 | 396 | 400 | 900 |
| *Reads mapped to genome* | 1853564 | 2078039 | 3181269 | 3217044 | 6978298 |
| *Reads mapped to target region* | 1045739 | 979315 | 1727709 | 1723036 | 3581817 |
| *Data mapped to target region (Mb)* | 85.05 | 79.43 | 141.25 | 140.75 | 252.71 |
| *Mean depth of target region (X)* | 61.58 | 57.52 | 102.28 | 101.92 | 169.78 |
| *Coverage of target region (%)* | 99.89 | 99.90 | 99.88 | 99.88 | 98.55 |
| *Average read length (bp)* | 89.89 | 89.89 | 89.89 | 89.93 | 89.84 |
| *Rate of nucleotide mismatch (%)* | 0.33 | 0.34 | 0.18 | 0.17 | 0.25 |
| *Fraction of target covered ≥4 X (%)* | 99.74 | 99.72 | 99.79 | 99.73 | 98.12 |
| *Fraction of target covered ≥10 X (%)* | 99.26 | 99.22 | 99.55 | 99.45 | 97.61 |
| *Fraction of target covered ≥20 X (%)* | 97.32 | 97.71 | 99.03 | 98.76 | 96.97 |
| *Capture specificity (%)* | 57.36 | 48.05 | 55.36 | 54.49 | 52.21 |
| *Reads mapped to flanking region* | 124142 | 129723 | 156865 | 160447 | 957551 |
| *Mean depth of flanking region (X)* | 16.91 | 16.95 | 24.19 | 24.56 | 80.05 |
| *Coverage of flanking region (%)* | 98.64 | 98.90 | 98.66 | 98.46 | 98.5 |
| *Fraction of flanking covered ≥4 X (%)* | 91.22 | 93.15 | 92.24 | 91.86 | 97.7 |
| *Fraction of flanking covered ≥10 X (%)* | 65.36 | 68.55 | 71.84 | 72.05 | 95.68 |
| *Fraction of flanking covered ≥20 X (%)* | 33.99 | 34.73 | 48.39 | 48.50 | 89.28 |
| *Fraction of unique mapped bases on or near target (%)* | 63.63 | 53.86 | 60.15 | 59.31 | 65.16 |
| *Duplication rate (%)* | 11.29 | 11.42 | 25.14 | 25.10 | 27.59 |
| *Mean depth of chrX (X)* | 31.72 | 57.17 | 108.72 | 112.18 | 170.21 |
| *GC rate (%)* | 43.87 | 43.46 | 43.44 | 42.45 | 45.36 |
| *Gender test result* | Female | Female | Female | Female | Female |
